# Gut mycobiota alteration contributes to the pathogenesis of *Pneumocystis* pneumonia

**DOI:** 10.1186/s12967-026-08153-7

**Published:** 2026-06-10

**Authors:** Yuxi Chen, Hanyujie Kang, Baolu Yang, Lin Feng, Renyang Tong, Zhiling Zhao, Lirong Liang, Xuyan Li, Xiaoyan Liu, Zhaohui Tong

**Affiliations:** 1 Department of Critical Care Medicine, Zhongnan Hospital of Wuhan University, Clinical Research Center of Hubei Critical Care Medicine, Wuhan, 430000 China; 2 Department of Respiratory and Critical Care Medicine, Beijing Institute of Respiratory Medicine and Beijing Chao-Yang Hospital, Capital Medical University, Beijing, 100020 China; 3 Medical Research Center, Beijing Institute of Respiratory Medicine and Beijing Chao-Yang Hospital, Capital Medical University, Beijing, 100020 China; 4 Beijing Key Laboratory of Multimodal Intelligent Diagnosis and Treatment System for Respiratory Diseases, Beijing Chao-Yang Hospital, Capital Medical University, Beijing, 100020 China

**Keywords:** *Pneumocystis* pneumonia, Gut mycobiome, Metabolomics

## Abstract

**Background:**

*Pneumocystis* is an opportunistic fungal pathogen that causes life-threatening pneumonia in immunocompromised hosts, with increasing incidence in HIV-negative individuals. Although the gut mycobiota has emerged as a critical regulator of distal immunity, its role in HIV-negative *Pneumocystis* pneumonia (PCP) remains entirely unexplored.

**Methods:**

We established a murine model of *Pneumocystis murina* infection and performed full-length internal transcribed spacer (ITS) sequencing to characterize longitudinal changes in gut fungal communities over five weeks. Untargeted metabolomics was conducted on plasma samples to identify systemic metabolic alterations. To investigate causality, gut fungal communities were depleted using fluconazole, and fecal microbiota transplantation (FMT) was performed in germ-free mice to assess the functional role of gut fungi in modulating pulmonary immune responses.

**Results:**

While α diversity of the gut mycobiota remained unchanged, β diversity analysis revealed significant structural alterations beginning week 3 (w3) post-infection, coinciding with peak pulmonary fungal burden. Linear discriminant analysis effect size identified *Purpureocillium lilacinum* and *Talaromyces verruculosus* as enriched opportunistic taxa. Untargeted metabolomics demonstrated marked metabolic reprogramming at w3, with significant perturbations in glycine, serine, and threonine metabolism, as well as the tricarboxylic acid cycle. Fluconazole-mediated depletion of gut fungi significantly increased pulmonary *Pneumocystis* burden and exacerbated lung inflammation, accompanied by reduced pulmonary Th1 cell responses. Critically, FMT from fluconazole-treated donors into germ-free mice recapitulated the exacerbated phenotype, confirming that gut fungal dysbiosis is sufficient to impair Th1-mediated antifungal immunity and worsen disease severity.

**Conclusions:**

This study establishes, for the first time, that gut fungal dysbiosis actively contributes to the pathogenesis of HIV-negative PCP via the gut-lung axis. Our findings reveal that commensal gut fungi support pulmonary Th1 immune responses essential for controlling PCP, and their disruption exacerbates disease. These results provide new insights into the gut mycobiota as a potential therapeutic target in PCP and caution against indiscriminate antifungal use in susceptible populations.

**Graphical abstract:**

**Figure:**
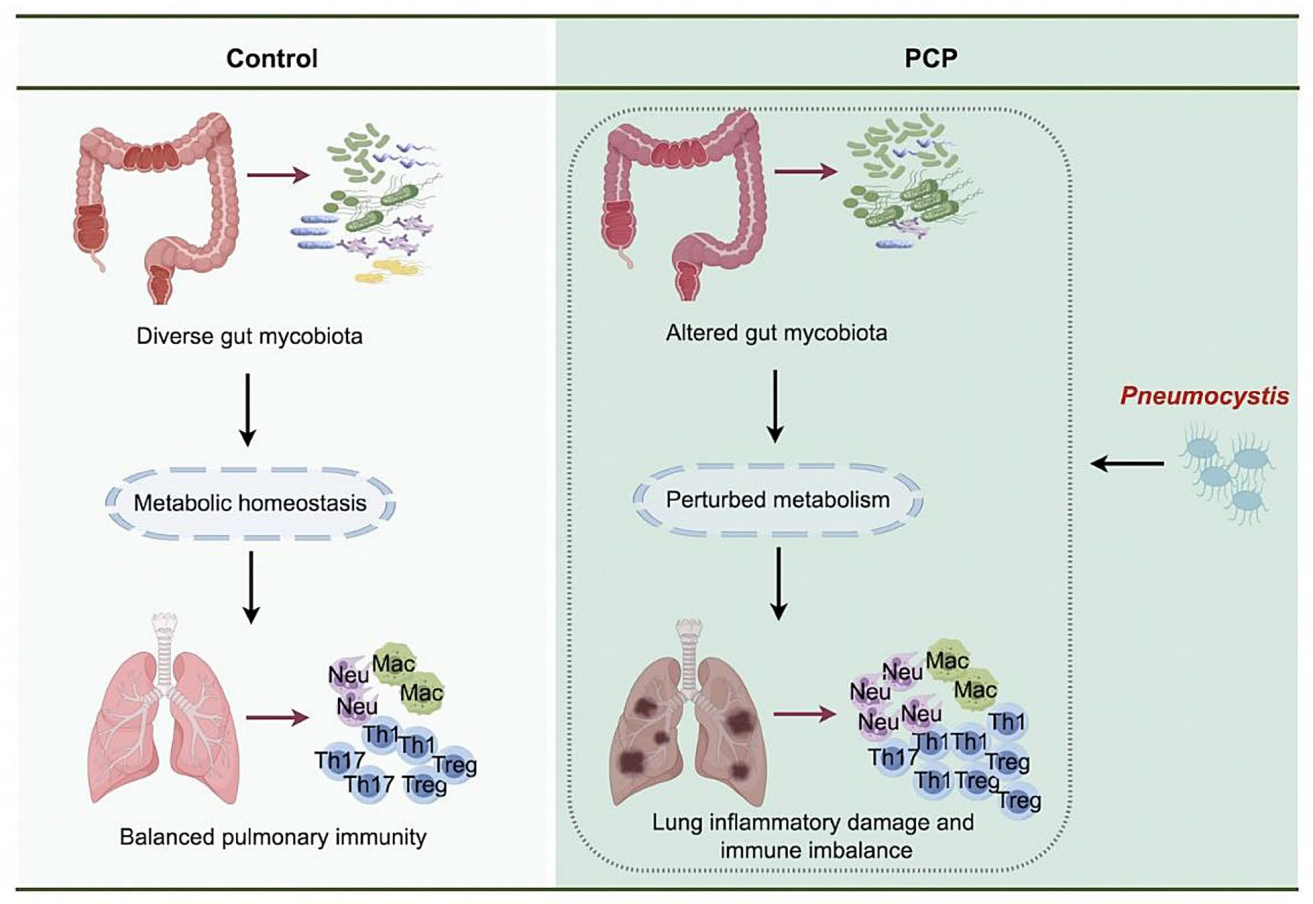
This graphical abstract illustrates the role of the gut mycobiota in HIV-negative PCP. In healthy controls, a diverse gut fungal community maintains metabolic homeostasis and balanced pulmonary immunity. During PCP, the gut mycobiota undergoes dysbiosis, accompanied by systemic metabolic perturbations and lung inflammatory damage with immune imbalance. These findings highlight a gut fungi-lung axis that critically influences disease outcomes in PCP

**Supplementary information:**

The online version contains supplementary material available at 10.1186/s12967-026-08153-7.

## Introduction

*Pneumocystis* is an opportunistic fungal pathogen of respiratory tract that can cause life-threatening severe pulmonary infections in immunosuppressed hosts. In recent years, with the wide clinical application of immunosuppressants and immunomodulators, the incidence of HIV-negative *Pneumocystis* pneumonia (PCP) has gradually increased [1]. PCP progresses more rapidly and carries higher risks of respiratory failure and mortality in HIV-negative individuals than in their HIV-positive counterparts [2]. Thus, there is an urgent need to identify new diagnostic and therapeutic targets for HIV-negative PCP.

The gut microbiome consists of bacteria, fungi, viruses, and archaea located in the intestinal tract. In recent years, many studies have focused on the role of the gut microbiota in pneumonia [3]. Kullberg et al. found that the characteristics of the intestinal bacteriome and virome were associated with the clinical outcomes of community-acquired pneumonia [4]. In addition, animal studies have described the protective role of the intestinal flora in host defense against *Streptococcus pneumoniae* infection [5].

Although gut mycobiota refers specifically to the fungal community residing in the intestinal tract, which comprises a relatively small part of the gut microbiome (0.1%-1%), accumulating evidence indicates that intestinal fungi play significant roles in various physiological and pathological processes [6–10]. A recent study found that gut fungi could ameliorate metabolic dysfunction-associated steatohepatitis in mouse models through a secondary metabolite-CerS6-ceramide axis [8]. In a study of *Klebsiella pneumoniae* infection, He et al. revealed that intestinal fungal dysregulation enhances alveolar macrophage-driven inflammation through Dectin-1 signaling, thereby exacerbating lung injury [11]. Intestinal fungal dysbiosis was also detected in both patients with COVID-19 and H1N1 influenza [12]. Despite this growing recognition of the gut-lung axis mediated by fungi, the role of intestinal fungi in PCP remains entirely unexplored. To date, studies on PCP and the microbiome have focused exclusively on bacterial communities in HIV-associated patients [13], leaving two critical questions unanswered: (1) whether and how the intestinal mycobiota is altered during HIV-negative PCP, and (2) whether gut fungi play a causal role in modulating pulmonary immune responses against PCP. Furthermore, while recent studies have highlighted that fungi can regulate distal organ immunity via their metabolites [7], whether such a “gut fungi-metabolite-lung immunity” axis operates in PCP has never been investigated.

Based on the above content, we hypothesized that the intestinal mycobiota is not merely altered during HIV-negative PCP, but actively contributes to the regulation of pulmonary immune responses against PCP through a gut-lung axis. Therefore, the present study examined the characteristic alterations in the intestinal fungi of *Pneumocystis*-infected mice and investigated the role of intestinal fungi in PCP-associated immunity, providing new perspectives and directions for uncovering potential therapeutic targets against PCP.

## Methods

### Ethics statement

All experimental protocols involving mice were performed in accordance with the guidelines of the US National Institutes of Health Guide for the Care and Use of Laboratory Animals and approved by the Welfare and Ethics Committee of Beijing Chao-Yang Hospital (25–2007). The experimental endpoints were defined as follows: a. body weight loss exceeding 20% of initial weight; b. inability to reach food or water; c. severe clinical signs (hunched posture, lethargy) not resolving within 24 hours. A total of 46 male C57BL/6J mice (8–10 weeks old) were used in the 0–5 w longitudinal experiment. Animals were randomly assigned to 6 groups, the specific group sizes were as follows: week 0 (w0), *n* = 8; w1, *n* = 7; w2, *n* = 7; w3, *n* = 8; w4, *n* = 8; w5, *n* = 8. For the intestinal fungal microbiota depletion experiment, animals were randomly divided into two groups (*n* = 6 per group). In the experiment comparing vehicle-treated uninfected and fluconazole-treated uninfected mice, animals were randomly assigned to each group (*n* = 6 per group). No animals reached these endpoints prematurely during the course of the experiment.

### Mice

C57BL/6J male mice (8 weeks old) were purchased from Beijing Vital River Laboratory Animal Technology Co., Ltd. (Beijing, China). The mice were housed under specific pathogen-free conditions at Beijing Chao-Yang Hospital. Germ-free male mice (8 weeks old) were purchased from GemPharmatech Co. Ltd. (Beijing, China), and were housed in plastic flexible film gnotobiotic isolators with high efficiency particulate air filter (HEPA) filters and with access to sterilized water and food ad libitum. All experiments containing germ-free mice were conducted in GemPharmatech Gnotobiotic Facility. The animals were kept under a 12-h/12-h light/dark cycle at a controlled temperature of 25 °C with unrestricted access to food and water.

### Fungal microbiota depletion

Mice were provided with autoclaved water supplemented with 0.5 mg/mL fluconazole (PHR1160, Sigma-Aldrich, St. Louis, MO, USA) for 5 days prior to the initiation of *Pneumocystis* inoculation [14–16]. Antifungal treatment was continued throughout the course of the experiments.

### Infection with *Pneumocystis murina*

*Pneumocystis murina* strains were acquired from the American Type Culture Collection (Manassas, VA, USA) and propagated in CB-17 SCID male mice (Beijing Vital River Laboratory Animal Technology Co., Ltd. Beijing, China) using established protocols. To generate PCP mouse models, each animal received an intratracheal inoculation of 1 × 10^6^ cysts suspended in 100 µL of sterile PBS. At designated time points, mice were euthanized under anesthesia following approved ethical guidelines.

### Fecal microbiota transplantation experiment

To obtain gut fungal communities with or without fluconazole-induced dysbiosis, donor mice were randomly assigned to two groups (*n* = 10 per group). The control group received autoclaved drinking water ad libitum, while the fluconazole-treated group received autoclaved water supplemented with 0.5 mg/mL fluconazole for 21 consecutive days. Fresh fecal pellets were collected from each donor group under sterile conditions, pooled, and immediately processed. Fecal samples were suspended in sterile anaerobic PBS at a concentration of 100 mg feces per 1 mL PBS, vortexed thoroughly for 5 min, allowed to settle for 10 min, and then centrifuged at 500 ×g for 3 min to remove large debris. The resulting supernatant, containing the fecal microbiota, was collected and used for transplantation.

Recipient mice (germ-free C57BL/6J male mice, 8 weeks old) were first infected with *Pneumocystis murina* via intratracheal inoculation (1 × 10^6^ cysts per mouse) as described above. Starting on day 3 post-infection, each recipient mouse received 200 μL of the fecal microbiota suspension from either control or fluconazole-treated donors by oral gavage (*n* = 6 per group). The FMT was administered once daily. At w3 post-infection, all recipient mice were euthanized, and lung tissues and blood samples were collected for subsequent analyses.

### Flow cytometry

All flow cytometric staining was performed as previously described [17]. Single cells from lung tissues were preincubated with Fc block (553141, Becton, Dickinson and Company, East Rutherford, NJ, USA) for 5 min and stained using Live/Dead dye in PBS, followed by extracellular staining in PBS at 4 °C. For intracellular detection of cytokines, cells were stimulated for 5 h at 37 °C, added with Cell Stimulation Cocktail at 2 ul/mL (00–4975-93, Thermo Fisher Scientific, Waltham, MA, USA). Cells were surface-stained with extracellular mAbs in PBS for  30 min at 4 °C. Cells were resuspended in a Foxp3/Transcription Factor Staining Buffer Set (00–5523-00, Thermo Fisher Scientific) and incubated with intracellular cytokine mAbs for 30 min at 4 °C. Cells were then resuspended in PBS for flow cytometric analysis (FACS Canto II, BD Biosciences, San Jose, CA, USA).

The following reagents and antibodies were utilized for flow cytometry: Ghost dye Red780 (13–0865-T100, Cytek Biosciences, Fremont, CA, USA), Fixable Viability Dye eFluor™ 506 (65–0866-14, Thermo Fisher Scientific), anti-CD45 (75–0451-U100, Cytek Biosciences), anti-CD3 (553066, Becton, Dickinson and Company), anti-CD4 (35–0042-U100, Cytek Biosciences), anti-CD11b (60–0112-U100, Cytek Biosciences), anti-Ly6G (551461, Becton, Dickinson and Company), anti-CD64 (139304, BioLegend, San Diego, CA, USA), anti-Mertk (12–5751-82, Thermo Fisher Scientific), anti-IFNγ (50–7311-U100, Cytek Biosciences), anti-IL17A (48–7177-82, Thermo Fisher Scientific), anti-Foxp3 (25–5773-82, Thermo Fisher Scientific), anti-CD86 (25–0862-82, Thermo Fisher Scientific) and anti-CD206 (565250, BD).

### RNA extraction and quantitative polymerase chain reaction (qPCR)

The middle lobe of the right lung was used to quantify the *Pneumocystis* burden by qPCR. RNA was extracted using TRIzol reagent (DP424, Tiangen, Beijing, China), quantified, and reverse-transcribed into cDNA using reverse transcriptase (RR047A, Takara, Shiga, Japan). The *Pneumocystis* burden was evaluated via TaqMan assays as previously reported (RR390A, Takara, Shiga, Japan).

### Hematoxylin and eosin (H&E) staining

Lung tissues were fixed in 4% paraformaldehyde and then embedded in paraffin. Following fixation, the tissues were dehydrated through a graded ethanol series and embedded in paraffin. Subsequently, paraffin-embedded tissues were sectioned at a thickness of 5 μm and stained with H&E for histological analysis.

### Stool sample collection and DNA extraction

Fresh fecal pellets were collected from mice at the designated time points (w0, *n* = 8; w1, *n* = 7; w2, *n* = 7; w3, *n* = 8; w4, *n* = 8; w5, *n* = 8). To minimize environmental contamination, mice were temporarily placed in sterile, empty cages without bedding, and stool samples were collected immediately after defecation using sterile forceps. Samples were then transferred into pre-labeled, DNase/RNase-free microcentrifuge tubes and stored at −80 °C until further processing. Total genomic DNA was extracted from the fecal samples by Oriental Yeekang (Beijing) Medicine Technology Co., Ltd. using a kit from Tiangen according to the manufacturer’s recommendations.

### Third-generation sequencing of the full-length internal transcribed spacer (ITS)

A full-length ITS-based amplicon covering the ITS1 and ITS2 variable regions was chosen as the amplification template, and ITS1F (5′-CTTGGTCATTTAGAGGAAGTAA-3′) and ITS4R (5′-TCCTCCGCTTATTGATATGC-3′) served as the upstream and downstream primers, respectively. PCR was performed in triplicate using a 20-μL reaction mixture containing 4 μL of 5× FastPfu Buffer (TransGen Biotech, Beijing, China), 2 μL of 2.5 mM dNTPs, 0.8 μL of each primer (5 μM), 0.4 μL of FastPfu Polymerase (TransGen Biotech), and 10 ng of template DNA. Amplicons were extracted from 2% agarose gels and purified using the AxyPrep DNA Gel Extraction Kit (Axygen Biosciences, Union City, CA, USA) according to the manufacturer’s instructions.

### 16S rDNA gene sequencing

Microbial DNA was extracted from mice stool samples using the E.Z.N.A.® Soil DNA Kit (Omega Bio-tek, Norcross, GA, U.S.) according to manufacturer’s protocols. The V1–V9 region of the bacteria 16S ribosomal RNA gene were amplified by PCR (95 °C for 2 min, followed by 27 cycles at 95 °C for 30 s, 55 °C for 30 s, and 72 °C for 60 s and a final extension at 72 °C for 5 min) using primers 27F 5′-AGRGTTYGATYMTGGCTCAG-3′ and 1492 R 5′-RGYTACCTTGTTACGACTT-3′, where barcode is an eight-base sequence unique to each sample (Pacific Biosciences, PN: 102–135-500). PCR reactions were performed in triplicate 20 μL mixture containing 4 μL of 5 × FastPfu Buffer, 2 μL of 2.5 mM dNTPs, 0.8 μL of each primer (5 μM), 0.4 μL of FastPfu Polymerase, and 10 ng of template DNA. Amplicons were extracted from 2% agarose gels and purified using the AxyPrep DNA Gel Extraction Kit (Axygen Biosciences, Union City, CA, U.S.) according to the manufacturer’s instructions.

### Processing of sequencing data

PacBio raw reads were processed using SMRT Link Analysis software version11.0 to obtain multiplexed circular consensus sequence reads with the following settings: minimum number of passes = 3 and minimum predicted accuracy = 0.99. Raw reads were processed through SMRT Portal to filter sequences for length (>1000 bp or < 1800 bp) and quality. Sequences were further filtered by removing barcode and primer sequences with the lima pipeline (Pacific Biosciences, https://lima.how/).

### Operational taxonomic unit (OTU) generation

OTUs were clustered with a 98.65% similarity cutoff using UPARSE (version 10, http://drive5.com/uparse/). The phylogenetic affiliation of each ITS rRNA gene sequence was analyzed using the uclust algorithm (https://github.com/topics/uclust) against the Silva (SSU138.1) ITS rRNA database (http://www.arb-silva.de) with a confidence threshold of 80%. To account for differences in sequencing depth across samples, the OTU abundance table was rarefied to the minimum sequencing depth among all samples.

### Relative abundance and α diversity

To visualize the microbial community structure, the top 10 most abundant taxa at each taxonomic level (phylum, class, order, family, genus, species) were selected, and their relative abundance distribution was plotted as a histogram using the ggplot2 package in R (The R Foundation for Statistical Computing, Vienna, Austria).

For α diversity analysis, the observed species, Chao1, and Shannon indices were computed using the vegan package (version 2.6–2) within R to evaluate the microbial community’s species richness, diversity, and uniformity, respectively, across samples.

### β diversity

Principal coordinated analysis (PCoA) was utilized to enhance the assessment and visualization of β diversity. The analysis utilized a precomputed unweighted UniFrac distance matrix, which quantifies phylogenetic dissimilarity between microbial communities based on the presence/absence of taxa. The resulting principal coordinates were graphically represented to assess intersample variation across experimental groups.

### Intergroup species difference analysis

To characterize fungal community differences among the six experimental groups, we employed linear discriminant analysis effect size (LEfSe) to detect taxa exhibiting significant abundance variations and highlight biologically relevant discriminative features. The Kruskal-Wallis rank-sum test was performed to examine the changes and dissimilarities among classes, followed by linear discriminant analysis (LDA) to determine the size effect of each distinctively abundant taxa.

### Metabolite extraction

Metabolites were extracted from plasma samples using methanol-assisted protein precipitation. The extracts were then analyzed using an ultrahigh-performance liquid chromatography system (Vanquish, Thermo Fisher Scientific) equipped with a UPLC BEH Amide column. Raw data were converted to the mzXML format using ProteoWizard and processed with the R package XCMS for peak detection, extraction, alignment, and integration. Peaks with a missing rate exceeding 50% across all samples were first filtered. Missing values were then imputed using the k-nearest neighbors algorithm. Subsequently, peak areas were normalized and corrected by support vector regression.

The remaining peaks were annotated by searching against an in-house metabolite database, supplemented by public databases, predictive libraries, and the metDNA method for comprehensive metabolite identification. Only metabolites with identification confidence score > 0.5 and coefficient of variation (CV) < 0.3 in quality control samples were retained. For metabolites detected in both the positive and negative ionization modes, the entry with the highest identification confidence score and lowest CV was selected for further analysis.

### Untargeted metabolomics

Unsupervised principal component analysis (PCA) was conducted using the prcomp function in R (version 4.0.2). Variable importance in projection (VIP) scores were calculated to evaluate the contribution of each metabolite to group separation. Metabolites with VIP > 1.5, |log2(fold change)| >1, and false discovery rate (FDR) <0.05 were considered significantly differentially abundant. This VIP threshold was selected based on established metabolomics studies [18, 19].

### Enzyme-linked immunosorbent assay (ELISA)

Plasma TNFα (CSB-E08054m, CUSABIO) was detected by the ELISA kits according to manufacturer’s protocols.

### Statistical analysis

Continuous variables were expressed as the mean ± standard error of the mean for normally distributed data or median (interquartile range) for non-normally distributed data. Intergroup comparisons among the six subgroups were performed using one-way ANOVA (for parametric data) or the Kruskal-Wallis test (for nonparametric data), followed by pairwise post hoc tests (Student’s *t*-test for normally distributed data or the Mann-Whitney *U* test for non-normally distributed data, as appropriate).

Spearman’s correlation analysis was employed to evaluate potential relationships between gut fungal taxa and the metabolites. All statistical analyses were performed using GraphPad Prism 9.0 (GraphPad Software, Boston, MA, USA).

## Results

### Characteristics of enteric fungi disturbance during progression of *Pneumocystis* infection in mice

**Fig. 1:**
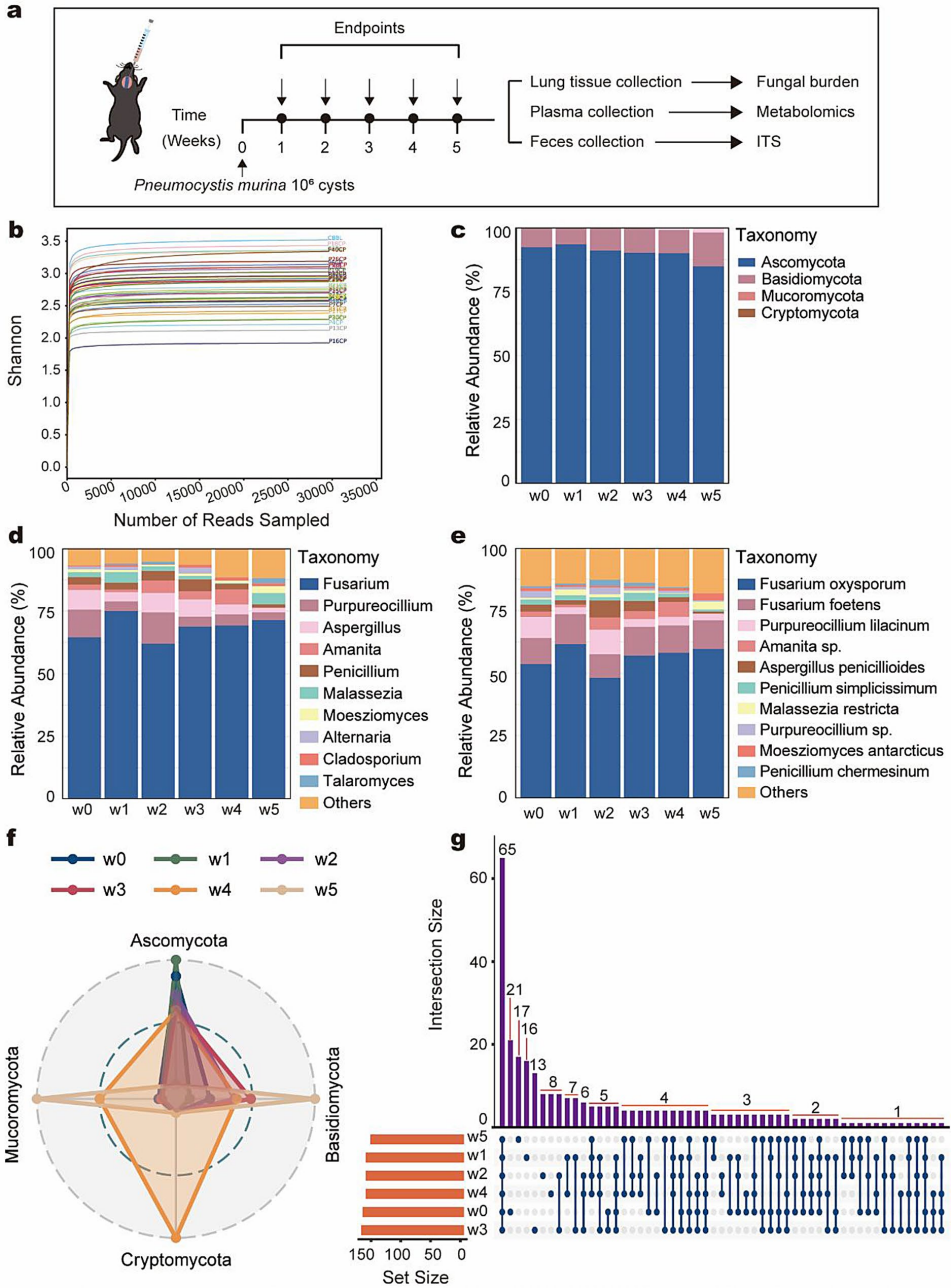
Fungal ecological characteristics in control and *Pneumocystis* pneumonia (PCP) progression subgroups. (**a**) Graphical depiction of the established murine PCP model. (**b**) Rarefaction curves of all samples. (**c**) Phylum-level relative abundance in various groups. (**d**) Genus-level relative abundance in various groups. (**e**) Species-level relative abundance in various groups. (**f**) Radar plot at the phylum level. (**g**) UpSetSet plot overlaps among the groups at the species level. Abbreviations: w0, week 0 (*n* = 8); w1, week1 (*n* = 7); w2, week2 (*n* = 7); w3, week3 (*n* = 8); w4, week4 (*n* = 8); w5, week5 (*n* = 8)

To elucidate the gut fungal community composition and diversity in response to PCP, a murine model of PCP was established and fecal samples were collected for third-generation sequencing of the full-length ITS at different time points post-infection (Fig. 1a). The sequencing depth was assessed using rarefaction curves (Fig. 1b), which demonstrated adequate coverage of fungal diversity, as all curves approached saturation, indicating that sufficient sequencing depth was achieved to capture the majority of fungal taxa present in each sample. Fungal community composition was assessed at the phylum, genus and species levels. At the phylum level (Fig. 1c), the fungal microbiota was predominantly composed of *Ascomycota* and *Basidiomycota*, which collectively represented the majority of sequences in all experimental groups. Further taxonomic classification at the genus level (Fig. 1d) identified *Fusarium*, *Purpureocillium*, *Aspergillus*, and *Amanita* as the predominant genera, and their prevalence was consistent across the groups. Notably, species-level analysis (Fig. 1e) identified *Fusarium oxysporum*, *F. foetens*, *Purpureocillium lilacinum*, and *Amanita sp.* as the most abundant species, suggesting their potential ecological or functional significance in the studied system. The radar plot (Fig. 1f) visually summarized the phylum-level distribution, highlighting key differences in fungal community structure among the groups. Meanwhile, overlaps of the six groups were depicted using an UpSet plot (Fig. 1g).

As shown in Figure S1, agarose gel electrophoresis following PCR amplification was performed on control sample and all experimental samples. The results showed no visible bands in the blank extraction control, whereas clear target bands were obtained for all experimental samples. These findings indicate that no detectable exogenous fungal DNA contamination was introduced during DNA extraction and PCR amplification, further supporting the reliability of the sequencing data.

### Ecological features of the fecal fungal flora in the PCP subgroups

**Fig. 2:**
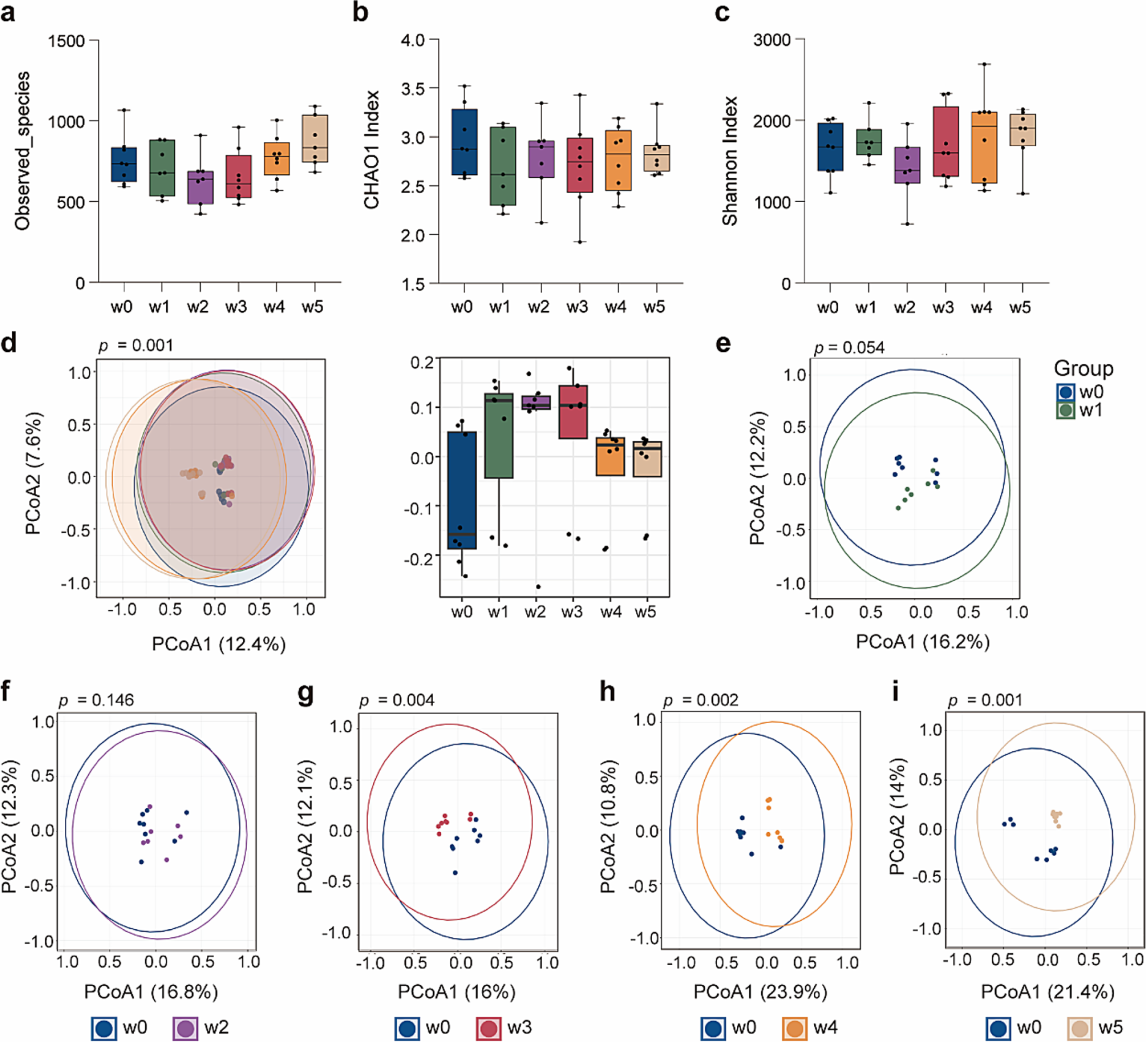
Comparative analysis of fungal diversity and discriminant factors in PCP. (**a-c**) comparative analysis of the observed species counts, Chao1 index, and Shannon index among the w0, w1, w2, w3, w4 and w5 PCP groups. Data are presented as the median and interquartile range. Differences among the six subgroups were assessed using the Kruskal–Wallis test. (**d**) PCoA plots illustrating unweighted UniFrac distances among all subgroups. (**e-i**) PCoA plots illustrating unweighted UniFrac distances among groups between different subgroups

Fungal community diversity was evaluated using multiple α diversity metrics, including the observed species (Fig. 2a), Chao1 (Fig. 2b), and Shannon indices (Fig. 2c). No significant differences were detected in these indices among the groups.

However, PCoA based on unweighted UniFrac distances revealed significant separation of mycobiome communities among the six subgroups (Fig. 2d). The first two principal coordinates accounted for 12.4% and 7.6% of the total variation, respectively. Collectively, these findings demonstrate that the PCP significantly altered the fungal community composition during its progression. The observed shifts suggest potential functional implications of mycobiome modulation in the PCP model.

To evaluate mycobiome community differences between PCP subgroups and controls, PCoA was performed using unweighted UniFrac distances. Although no significant differences were observed between the week 0 (w0) and w1 (*p* = 0.054), and w0 and w2 (*p* = 0.146), statistically significant compositional changes emerged from the week 3 onward (w0 vs w3: *p* = 0.004, w0 vs w4: *p* = 0.002, w0 vs w5: *p* = 0.001; Figs. 2e–2i).

**Fig. 3:**
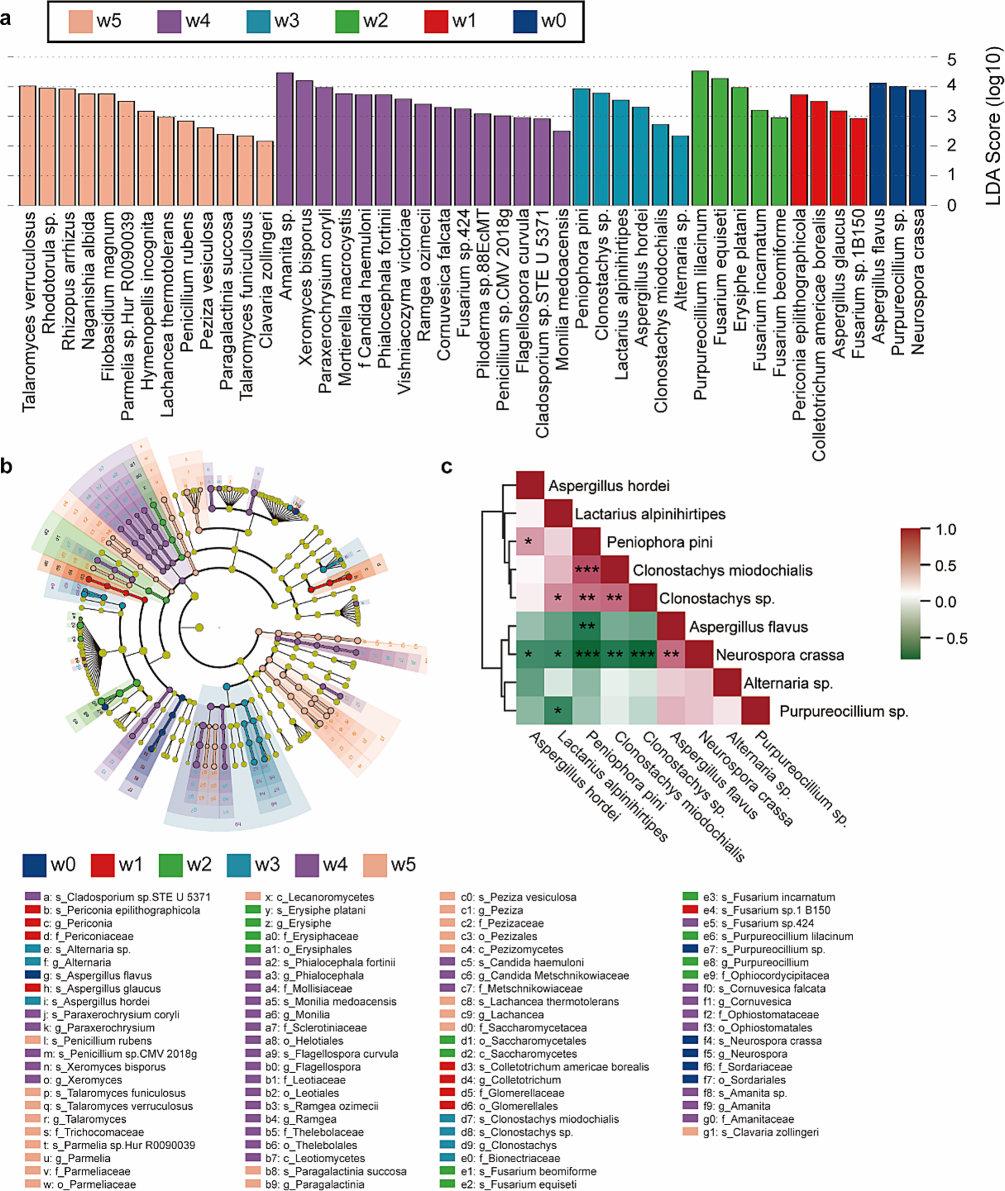
Fungal community differential analysis and correlation network. (**a**) LEfSe across different fungal taxon levels (LDA score > 2, *p* < 0.05). (**b**) Cladogram of differential taxa from class to species displaying taxonomic relationships and relative abundances across different levels. (**c**) Spearman’s’s correlations between fungal taxa from the w0 and w3 subgroups at the species level. Red and blue denote positive and negative correlations, respectively. The sizes of circles are proportional to the absolute correlation coefficient magnitude. *FDR < 0.2, **FDR < 0.1, *** FDR < 0.05

To determine which taxa exhibited significant variations across classification levels in different groups, we performed LEfSe. The results identified 46 differentially abundant species (Fig. 3a and 3b). The corresponding LEfSe results at the phylum and genus levels are presented in the supplementary figures (Fig. S2a-b). LEfSe revealed that the fungal community in the PCP subgroups was characterized by the significant enrichment of several taxa with potential pathogenic and stress-tolerant traits. Most notably, *Purpureocillium lilacinum*, a known opportunistic pathogen [20], emerged as the top fungal biomarker based on the LDA score. The disease groups also displayed a marked increase in the abundance of *Talaromyces verruculosus*, a prolific producer of secondary metabolites and enzymes [21]. The co-enrichment of these fungi suggests a dysbiotic state favoring organisms with pathogenic potential.

Spearman’s correlation analysis was performed to assess pairwise associations among differentially abundant fungal species. The results revealed significant interconnections within the fungal community, with multiple taxa exhibiting strong co-occurrence patterns, suggesting potential ecological interactions or niche competition among these taxa (Fig. 3c).

### Mice infected with *Pneumocystis* exhibited the heaviest fungal load and significant changes in the gut mycobiome at w3 post-infection

**Fig. 4:**
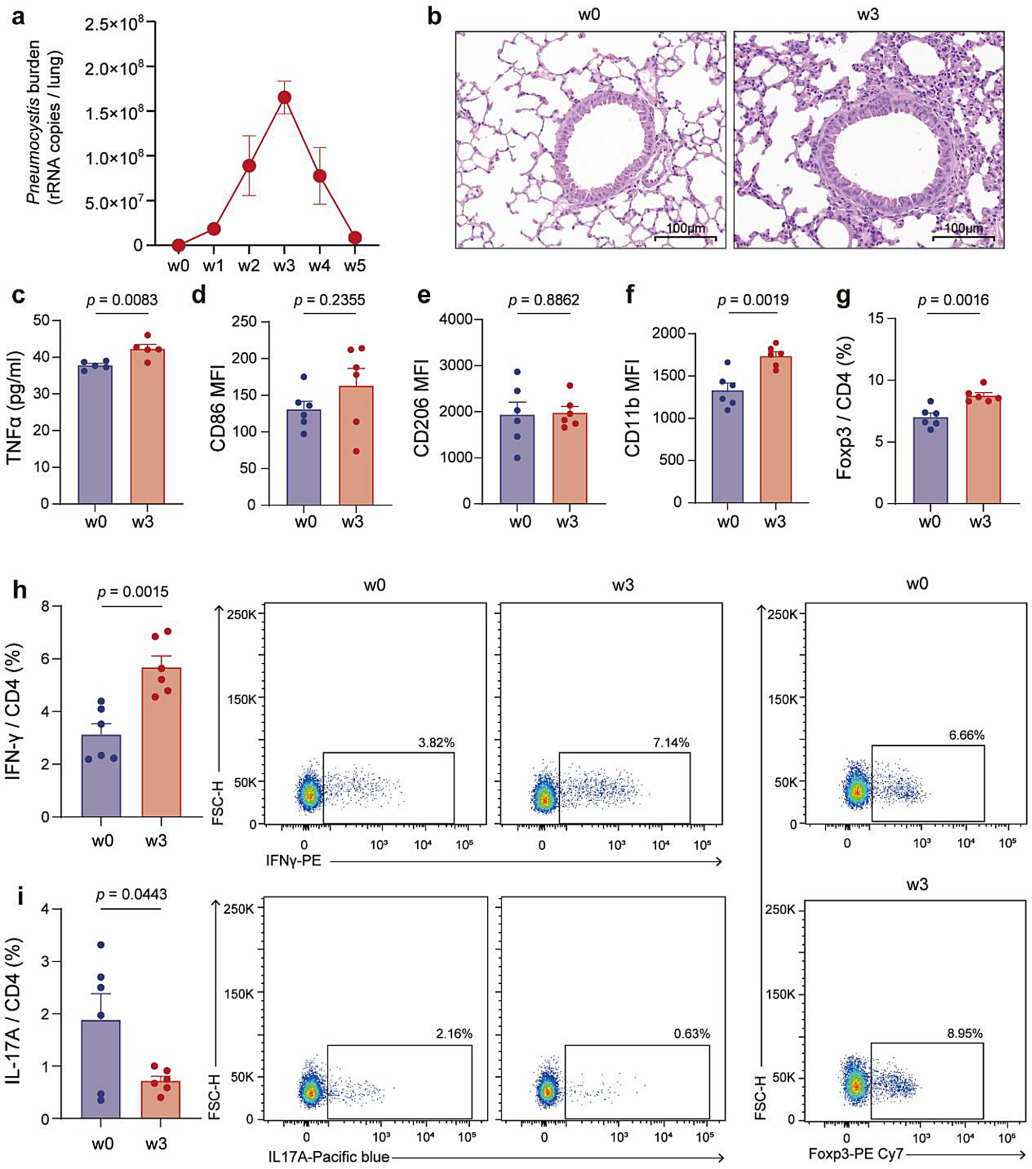
PCP kinetics and pulmonary responses. (**a**) Mice were intratracheally inoculated with *Pneumocystis*, and the fungal burden was longitudinally monitored over a 5-week period. (**b**) Representative lung H&E images from the w0 and w3 mice. Scale bars represent 100 μm. (**c**) ELISA result of TNFα in mice plasma from the w0 and w3 mice. (**d-e**) MFI of CD86 and CD206 on macrophages in the w0 and w3 mice. (**f**) MFI of CD11b on neutrophils in the w0 and w3 mice. (**g-i**) proportion of Tregs (**g**), Th1 (**j**) and Th17 (**i**) among pulmonary CD4^+^ T cells in the w0 and w3 mice. *n* = 4–6/group

The dynamic progression of PCP was characterized by a time-dependent increase in the pulmonary fungal burden, which peaked at w3 post-infection before gradually declining (Fig. 4a). Histopathological analysis demonstrated corresponding structural alterations, as H&E staining highlighted marked thickening of the alveolar septa and substantial inflammatory infiltration at w3 post-infection compared with the findings at baseline (Fig. 4b).

To investigate the underlying immune alterations, flow cytometry was performed. Representative flow cytometry gating strategies are shown in Figure S3 & S4. No significant differences were observed in the expression levels of M1 and M2 markers between the w0 and w3 groups. (Figs. 4d & 4e). Concurrently, neutrophils exhibited a markedly elevated mean fluorescence intensity (MFI) of CD11b in the w3 group (Fig. 4f), a canonical marker of neutrophil activation and a key integrin mediating adhesion and migration. These findings demonstrate that PCP potently polarizes neutrophils towards an activated, pro-inflammatory phenotype, suggesting their active involvement in the pathogenesis of pulmonary inflammation and injury. Given the pivotal role of CD4^+^ T cells in host defense against *Pneumocystis*, we comprehensively assessed the changes in each functional subgroup of CD4^+^ T cells within the pulmonary compartment. The frequency of IFNγ-producing Th1 cells and Foxp3^+^ regulatory T cells (Tregs) were markedly elevated (Figs. 4g & 4h). Conversely, we observed a significant reduction in the frequencies of IL17A-producing Th17 cells within the CD4^+^ compartment (Fig. 4i). This distinct immunological phenotype, characterized by a concurrent increase in Th1 and Treg cells and a decrease in Th17 populations, indicates that PCP drives a profound shift in the CD4^+^ T cell landscape towards a Th1-dominant state. Although the proportion of Tregs was significantly increased after infection, the compensatory upregulation of Tregs was insufficient to effectively control pulmonary pathological damage.

### Integrated gut-lung axis modulates PCP through metabolic reprogramming and fungi-metabolite crosstalk

**Fig. 5:**
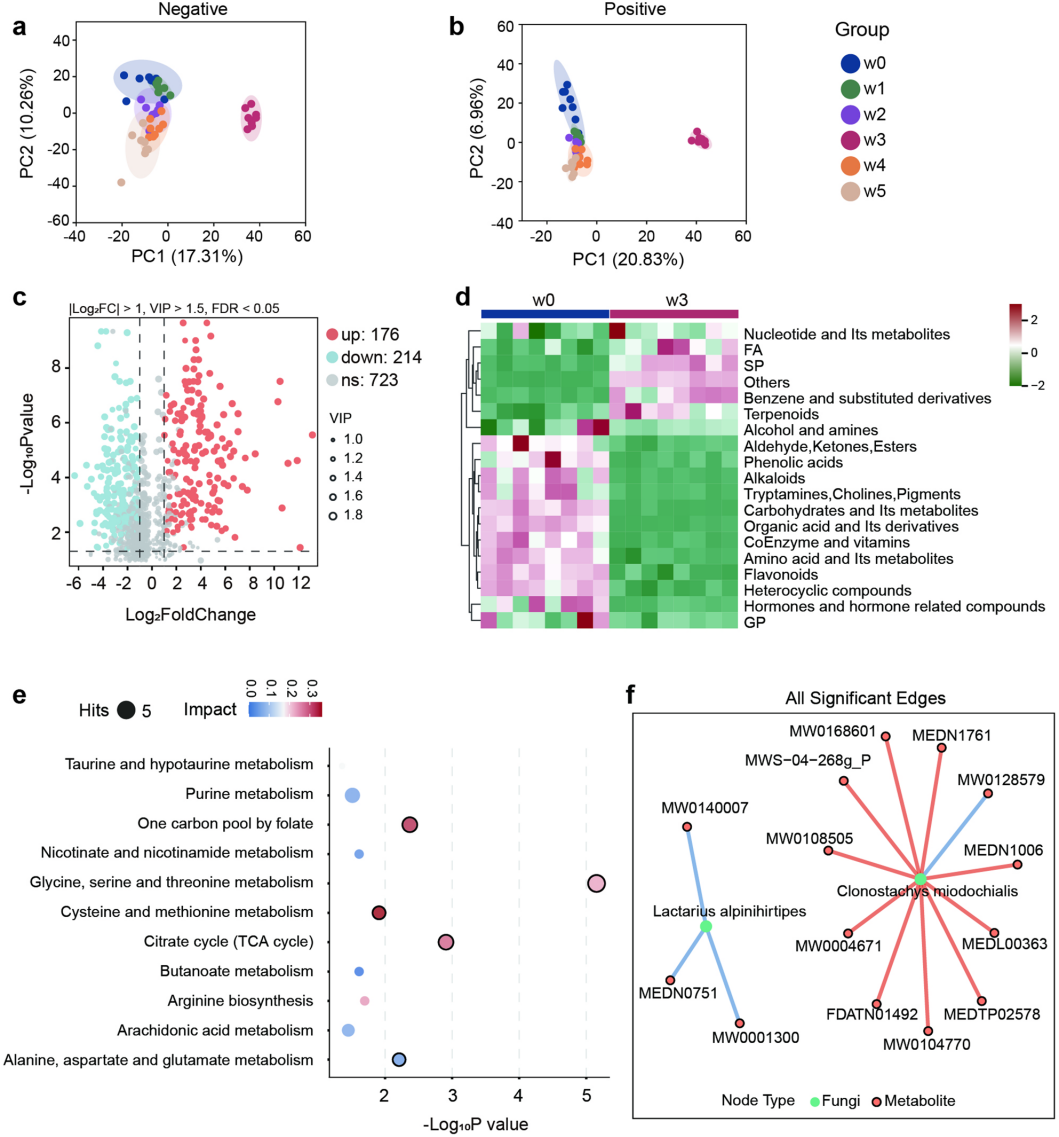
Alteration of plasma metabolites in stages of PCP progression. (**a-b**) Score plot of the PCA model in the negative and positive ion modes. (**c**) Volcano plot displaying significantly altered metabolites (w3 vs. w0). Red and blue denote significantly enriched and suppressed metabolites, respectively (VIP>1.5, *P* < 0.05, |log2(fold change)| > 1). (**d**) Hierarchical clustering heatmap of differential metabolites between the two subgroups. (**e**) Metabolic pathways enriched by differential metabolites in (d), analyzed using MetaboAnalyst. Pathways circled in the plot have an FDR < 0.2. (**f**) Original fungus-metabolite association network constructed using all significant edges (FDR < 0.2). Green nodes represent fungal taxa, orange nodes represent metabolites, red edges indicate positive correlations, and blue edges indicate negative correlations

Intestinal fungi can influence the physiological and pathological processes of the host through their metabolites [22, 23]. Therefore, we next investigated the changes in metabolite levels in the plasma of mice with PCP and their correlation with intestinal fungi by untargeted metabolomics. In both the positive and negative ionization modes, Principal component analysis (PCA) revealed clear separation among experimental groups, indicating substantial metabolic differences. Notably, significant metabolic divergence was observed in the w3 subgroup versus the other subgroups, demonstrating clear separation in the PCA score plots (Fig. 5a and 5b). To further characterize the metabolic alterations in the w3 group, we performed a comparison with the w0 group. Volcano plot analysis revealed 176 significantly enriched and 214 significantly downregulated metabolites at w3 (VIP > 1.5, FDR < 0.05, |log2 (fold change) >1|; Fig. 5c), according to the criteria described in the Methods section. Unsupervised clustering of these differential metabolites demonstrated pronounced subgroup segregation, with distinct enrichment patterns in lipid species including fatty acylsand sphingolipids, which exhibited consistently increased abundance at w3 (Fig. 5d).

To further investigate the possible metabolic pathways, we performed pathway enrichment analysis using Metaboanalyst (https://www.metaboanalyst.ca/). Notably, after adjustment for multiple comparisons, some pathways are disturbed, such as the Glycine, serine, threonine metabolism pathway and the Citrate cycle (TCA cycle), implicating dysregulated amino acid metabolism and energy homeostasis in the pathogenesis of PCP (Fig. 5e). To investigate potential associations between plasma metabolites and gut fungal taxa, we constructed correlation networks. Initial correlation analysis identified a set of significant fungus-metabolite associations, visualized in an unfiltered network (Fig. 5f). To assess the reliability of fungi-metabolite correlations, we performed bootstrap stability analysis (1,000 iterations) and retained only edges with ≥70% bootstrap stability (Fig. S5a-b). The resulting stable network (Fig. S5c) provides a robust representation of fungi-metabolite associations during PCP progression.

### Depletion of gut fungal communities increased the pulmonary *Pneumocystis* burden and exacerbated lung inflammation

**Fig. 6:**
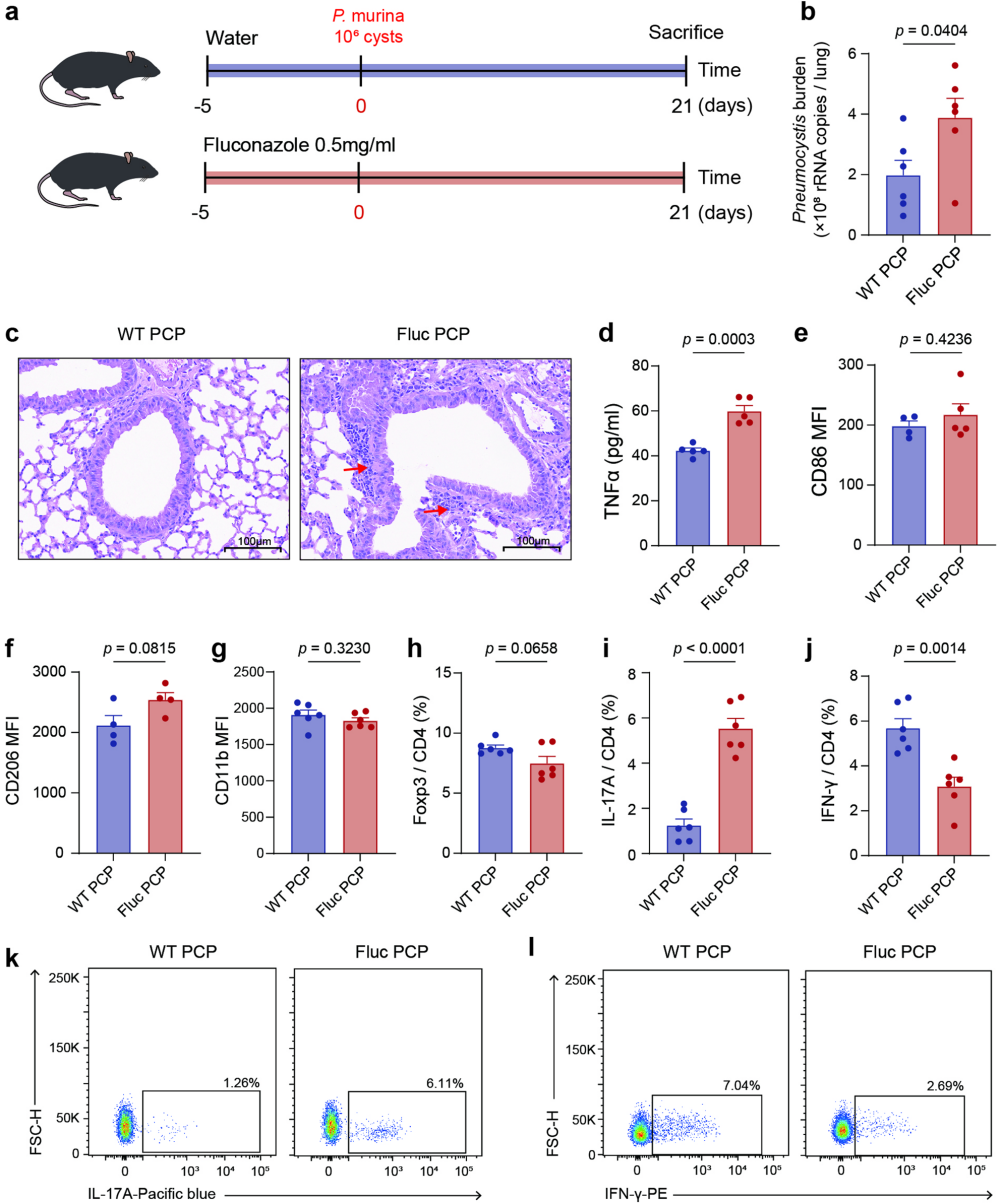
Impact of fluconazole on the PCP burden and pulmonary inflammation. (**a**) Schematic view of the infection schedule and overall study design. (**b**) The *Pneumocystis* burden was detected by qPCR in the untreated (WT PCP) and fluconazole-treated mice (Fluc PCP) at w3 post-infection. (**c**) Representative H&E images. Insets present magnified areas of cell infiltration and inflammation. Scale bars represent 100 μm. (**d**) ELISA result of TNFα in mice plasma from the WT PCP and Fluc PCP mice at w3 post-infection. (**e-f**) MFI of CD86 and CD206 on macrophages in the WT PCP and Fluc PCP mice at w3 post-infection. (**g**) MFI of CD11b on neutrophils in the WT PCP and Fluc PCP mice at w3 post-infection. (**h-j**) Proportion of Tregs, Th1 and Th17 among pulmonary CD4⁺ T cells in the WT PCP and Fluc PCP mice at w3 post-infection. *n* = 4-6 per group. (**k-l**) Representative flow cytometric plots of Th1 (live CD3^+^ CD4^+^ IFN-γ^+^) and Th17 (live CD3^+^ CD4^+^ IL-17A^+^) from WT PCP and Fluc PCP mice at w3 post-infection

To investigate the effect of intestinal fungi on the immune response to PCP, mice were pretreated with the antifungal drug fluconazole at 0.5 mg/mL for 5 days in their drinking water before intratracheal injection of *Pneumocystis murina* at a dose of 1 × 10^6^ cysts (Fig. 6a). At w3 post-infection, quantitative analysis of the pulmonary fungal burden was performed to evaluate *Pneumocystis* clearance (Fig. 6b) and flow cytometry was utilized to detect the pulmonary immune environment (Fig. 6e–j).

Depletion of the intestinal mycobiota significantly exacerbated pulmonary fungal burden in *Pneumocystis*-infected mice (Fig. 6b), indicating a protective role of commensal gut fungi against respiratory fungal infection. H&E staining further demonstrated enhanced inflammatory infiltration and marked thickening of alveolar septa (Fig. 6c), consistent with aggravated lung pathology. Plasma TNFα level was significantly elevated in Fluconazole-treated PCP (Fluc PCP) group (Fig. 6d). The aggravated infection phenotype was accompanied by substantial remodeling of the pulmonary immune landscape, as revealed by comprehensive flow cytometric analysis (Fig. 6e–j). Specifically, we documented a pronounced reduction in pulmonary Th1 cells coupled with substantial Th17 cells increasing in Fluc PCP. Collectively, these findings establish that intestinal fungal communities critically modulate host immunity during PCP. The observed Th1 cells reduction likely compromises effective fungal clearance mechanisms, whereas the robust Th17 cells expansion suggests dysregulated inflammatory responses. This dual defect in both pathogen elimination and inflammation control appears to underlie the worsened disease severity following mycobiota disruption.

To evaluate the direct immunomodulatory effects of fluconazole, uninfected control mice were treated with or without fluconazole (Fig. S6a). H&E staining revealed no histopathological abnormalities in the lungs (Fig. S6b), and levels of inflammatory cytokines remained comparable between the two groups (Fig. S6c). Flow cytometric analysis showed that fluconazole-treated mice exhibited significantly increased proportions of Th1 and Th17 cells among CD4^+^ T cells compared to controls, whereas other immune cell subsets remained unchanged (Fig. S6d-h). These data suggest that while fluconazole selectively modulates Th1 and Th17 responses in healthy mice, these changes are insufficient to drive pulmonary inflammation in the absence of an infectious challenge. By contrast, under co-infection conditions, more substantial immune alterations were evident, suggesting that the immunomodulatory effects of fluconazole are amplified in the context of infection.

To determine whether fluconazole treatment perturbs the bacterial community, we performed 16S rRNA gene amplicon sequencing and ITS sequencing on the fecal samples from Fluc PCP mice and vehicle-treated PCP (WT PCP) mice. Comparative analyses revealed the following: For gut bacterial community, no significant differences in α-diversity (Shannon, Chao1, observed species) or β-diversity (PERMANOVA analysis) were detected between WT PCP and Fluc PCP group. In contrast, fluconazole treatment significantly reduced fungal observed species (α-diversity) and induced clear separation in β-diversity (PERMANOVA analysis) (Fig. S7). These results demonstrate that fluconazole intervention has a much stronger impact on the gut fungal community than on the bacterial community. This finding is consistent with previously published studies [24].

### Gut fungal dysbiosis is causally linked to the *Pneumocystis* burden and pulmonary inflammation via fecal microbiota transplantation

**Fig. 7:**
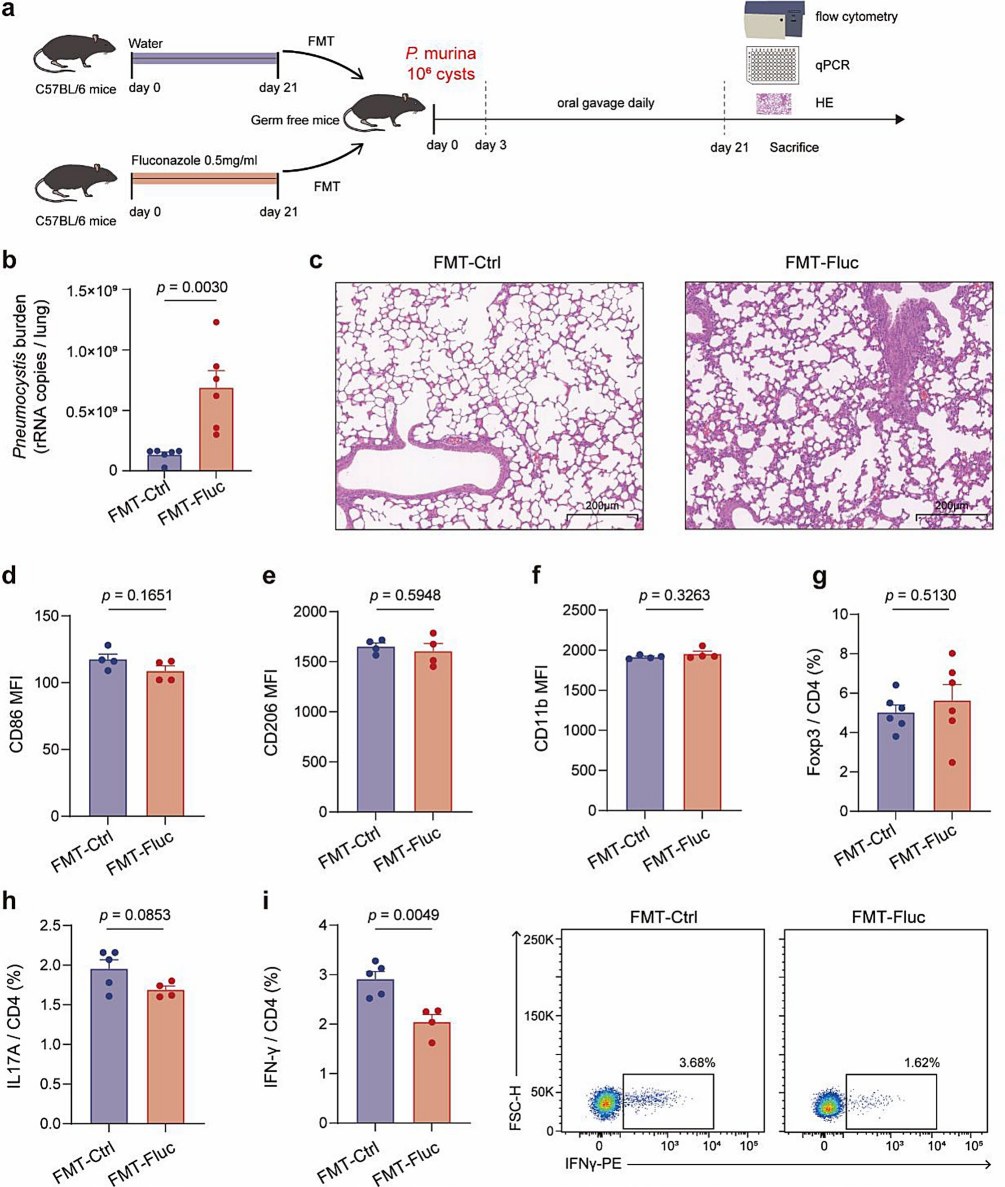
Fecal microbiota transplantation (FMT) from fluconazole-treated donors exacerbates PCP in germ-free (GF) mice. (**a**) Schematic overview of the experimental design. GF mice were infected with *Pneumocystis* murina, followed by FMT via gavage using donor material from fluconazole-treated (FMT-Fluc group) or control (FMT-Ctrl group) mice. Recipient mice were euthanized at the indicated time points for analysis of pulmonary *Pneumocystis* burden, lung inflammation, and immune cell profiles. (**b**) The *Pneumocystis* burden was measured by qPCR in the FMT-Ctrl and FMT-Fluc recipients at week3 (w3) post-infection. (**c**) Representative H&E staining of lung sections from FMT-Ctrl and FMT-Fluc recipients at w3 post-infection. Scale bars, 200 μm. (**d-e**) Mean fluorescence intensity (MFI) of CD86 and CD206 on macrophages in the FMT-Ctrl and FMT-Fluc recipients at w3 post-infection. (**f**) MFI of CD11b on neutrophils in the FMT-Ctrl and FMT-Fluc recipients at w3 post-infection. (**g-i**) Proportion of Tregs, Th1 and Th17 among pulmonary CD4^+^ T cells in the FMT-Ctrl and FMT-Fluc recipients at w3 post-infection. *n* = 4–6 per group

To investigate whether the gut fungal communities mediate the effects of fluconazole on PCP, we performed FMT experiments (Fig. 7a). Germ-free recipient mice were infected with *Pneumocystis* and then colonized with fecal mycobiome obtained from donor mice that were either untreated or treated with fluconazole. The recipients were subsequently evaluated for fungal burden, lung inflammation, and immune cell profiles.

Strikingly, germ-free mice colonized with the dysbiotic fecal mycobiota from fluconazole-treated donors (FMT-Fluc) exhibited significantly higher pulmonary *Pneumocystis* burden compared to recipients of control fecal mycobiota (FMT-Ctrl) (Fig. 7b). H&E staining revealed more severe lung tissue damage, including alveolar wall thickening and inflammatory foci, in the FMT-Fluc group (Fig. 7c). This indicates that the altered gut fungal configuration is sufficient to confer susceptibility to PCP, and the elevated *Pneumocystis* load may contribute to the exacerbation of pulmonary inflammation. Flow cytometry analysis revealed immune cell alterations in the lungs of PCP mice in the FMT experiment (Fig. 7d–i). Consistent with the increased fungal burden, FMT-Fluc group exhibited a reduction in antifungal Th1 cells within the lung tissues. (Fig. 7i). The FMT experiments provide direct causal evidence that gut fungal dysbiosis, induced by fluconazole treatment, is sufficient to exacerbate PCP by modulating pulmonary immune responses. These findings establish a functional gut-lung axis mediated by gut fungi in the context of PCP.

## Discussion

In the present study, we revealed for the first time, to our knowledge, that PCP can induce dysbiosis in the gut fungal community, which became most pronounced at the w3 time point, coinciding with the peak *Pneumocystis* burden. Meanwhile, plasma metabolite levels displayed prominent changes at w3 post-infection, and these changes were significantly associated with specific gut fungal species. Importantly, fluconazole treatment disrupted intestinal fungal homeostasis, leading to a decline in the protective Th1 immunity response in the lungs, a compensatory increase in Th17 cells, and exacerbated *Pneumocystis* burden and pulmonary inflammation. FMT experiments confirmed that gut fungal dysbiosis is sufficient to exacerbate PCP by suppressing the Th1 cell response.

Although the gut mycobiota constitutes a marginal proportion of the intestinal microbiome, its role in host immune regulation is increasingly recognized [25]. In the present study, significant separation in β diversity of the gut mycobiota was noted from w3 post-infection in mice with PCP, whereas α diversity remained unaltered. This indicates that structural rearrangement, rather than a change in species richness, represents the primary characteristic of PCP-associated gut fungal dysbiosis. This pattern mirrors the gut fungal dysbiosis previously observed in coronary artery disease [9], suggesting that disease-specific structural alterations of fungal communities may exist across distinct pathological conditions.

Our results revealed that the abundance of *Aspergillus penicillioides*, *Purpureocillium sp.*, and *Penicillium chermesinum* increased concomitantly with the rising fungal load and subsequently declined as the fungal burden returned to baseline levels. In the PCP mouse model, pulmonary inflammation distally disrupted gut homeostasis via the gut-lung axis. This led to impaired intestinal barrier function, as has been observed in other models of inflammation and infection [26], facilitating the proliferation of opportunistic pathobionts (e.g. *Alternaria* [27], *Aspergillus* [28]). Ultimately, this dysbiotic gut mycobiota might have systemically influenced the host’s immune competence through the release of immunogenic compounds, thereby adversely affecting the progression and resolution of the primary pulmonary PCP, as supported by the established role of the gut-lung axis in immune regulation [5, 29, 30].

The mechanisms by which gut fungi regulate distal organ immunity have been progressively elucidated in recent years [31]. Studies have shown that gut fungi can influence the immune status of distal organs through their metabolites, which enter the circulatory system [8]. The plasma metabolomic profile of mice with PCP was markedly remodeled at the peak of infection, characterized by significantly elevated levels of lipid metabolites and disturbances in metabolic pathways, such as the glycine, serine and threonine metabolism, and the TCA cycle pathways. Glycine, serine, and threonine metabolism participates in glutathione synthesis and one-carbon metabolism, thereby influencing T cell activation and proliferation [32]. The TCA cycle is a central pathway of energy metabolism, and its perturbation directly reflects the metabolic reprogramming of immune cells [33]. Li et al. demonstrated that modulation of the gut microbiota by FMT influences pulmonary Th17-mediated inflammatory responses through mechanisms involving alterations in metabolites such as short-chain fatty acids [34]. Similarly, de Almeida et al. reported that oral administration of the probiotic *Lactobacillus delbrueckii* induces intestinal expansion of FoxP3^+^ regulatory T cells and remotely regulates pulmonary immune responses against *Aspergillus fumigatus* via metabolites [35]. These findings are highly consistent with the metabolic pathway perturbations observed in our study, collectively supporting the existence of a gut microbiome-metabolite-lung immune axis. Pathway enrichment analysis revealed perturbations in amino acid metabolism and energy metabolic pathways in PCP, consistent with the known metabolic reprogramming during fungal infections. These pathways are critical for maintaining cellular homeostasis and host-microbe interactions, suggesting their potential involvement in mediating host responses to PCP. Correlation analysis between differential metabolites and microbiota in the w3 group provides preliminary clues about possible functional links between microbial community shifts and metabolic alterations in PCP. Future studies focusing on isolating and characterizing *Clonostachys miodochialis* and *Lactarius alpinihirtipes* and identify and profile metabolites derived from these two species, along with in vitro and in vivo assays to evaluate their impact on *Pneumocystis* colonization and host immune function, will be essential to validate and expand on these preliminary findings. We acknowledge alternative interpretations of the observed fungi-metabolite correlations. These associations may reflect reverse causation, whereby infection driven immune or metabolic changes promote the outgrowth of certain fungi rather than these fungi actively modulating host metabolism. Additionally, some detected fungi may represent transient dietary or environmental spores rather than stable gut colonizers. Finally, elevated plasma metabolites could arise from systemic stress responses secondary to pulmonary infection, independent of gut fungal activity. Distinguishing these possibilities will require targeted intervention studies with defined fungal consortia or purified metabolites.

The gut mycobiota influences immune responses in the lungs via the gut-lung axis, and alterations to the gut mycobiota can disrupt airway immunity [10, 29, 36]. These findings underscore the importance of the gut mycobiota in maintaining immune homeostasis, particularly during pulmonary infection, which represents a key target of opportunity. The central role of T cells in PCP has been well established [37]. A recent review systematically summarized the functional heterogeneity of T-cell subsets in PCP, clearly indicating that the proportion of Th1 cells in the lungs is significantly elevated after infection, and that low Th1 cells levels are associated with poor prognosis [38]. In our study, we observed a marked increase in pulmonary Th1 cells proportions in PCP mice, which is consistent with previous studies. More importantly, our results demonstrate that depletion of gut fungi with fluconazole led to a significant reduction in pulmonary Th1 cells, accompanied by increased fungal burden and exacerbated lung inflammation, potentially explains the worsened disease outcome. These findings underscore the necessity of the gut mycobiota for sustaining the pulmonary Th1 immune response and establish the intestinal mycobiota as a critical remote regulator of balanced immune responses in the lungs.

Although fluconazole acts primarily as an antifungal agent, its effects in vivo may involve both potential off-target effects on other microbial constituents and indirect consequences of fungal depletion. Regarding off-target effects, fluconazole may exert unintended effects on gut bacteria. To address this, we performed 16S rRNA gene sequencing to evaluate the impact of fluconazole on the gut bacterial community. Our data confirmed minimal bacterial disruption, supporting that the observed immune changes are primarily attributable to fungal perturbation rather than broad-spectrum antimicrobial off-target effects. Regarding indirect effects, we observed that fluconazole treatment in uninfected mice led to a modest but significant increase in Th1 and Th17 cell proportions. This immune modulation likely reflects the indirect consequences of gut fungal depletion rather than direct drug-immune cell interactions, given that fluconazole has minimal known direct immunomodulatory activity. In uninfected mice, these changes were insufficient to trigger overt pulmonary inflammation. However, during PCP, such indirect effects were amplified in the context of inflammatory stress, contributing to exacerbated lung inflammation and impaired pathogen clearance. We therefore conclude that fluconazole-associated worsening of PCP is driven primarily by depletion of gut fungi and the consequent indirect immune modulation, rather than by non-specific off-target effects on bacteria or direct immunotoxicity.

The causal relationship between gut fungal dysbiosis and PCP severity was definitively established through our FMT experiments in germ-free mice. In this study, after transplantation of fluconazole-induced dysbiotic fungal communities into germ-free mice, the recipient mice exhibited a PCP-exacerbated phenotype, characterized by increased fungal burden, aggravated inflammation, and reduced Th1 cells. This germ-free FMT approach, considered the gold standard for establishing microbiota-host causal relationships, provides compelling evidence that gut fungi actively participate in the pathogenesis of PCP through the gut-lung axis. A recent study also showed that gut dysbiosis induced by antibiotics can increase susceptibility to fungal pneumonia through the gut-lung axis, involving mechanisms such as pulmonary metabolic disturbances and altered inflammatory responses [39]. The mechanisms by which gut fungi participate in disease pathogenesis may involve multiple interconnected pathways [40]. First, fungal cell wall components are recognized by pattern recognition receptors such as C-type lectin receptors (Dectin-1, Dectin-2, Mincle), triggering Syk-dependent signaling and promoting pro-inflammatory cytokine production [41]. Second, gut fungal dysbiosis can compromise intestinal barrier integrity, either through virulence factors or by disrupting IL-22 mediated epithelial repair, thereby facilitating local and systemic inflammation [42]. Third, cross-kingdom interactions between fungi and bacteria, such as metabolic competition or shifts from competitive to synergistic networks, may reshape the microbial ecosystem and influence host immune homeostasis [9, 10]. Finally, fungal derived metabolites (e.g., FXR agonists) and diet-fungus-host metabolic axes may directly modulate host physiology [43]. Together, these mechanisms support the concept that gut fungi actively contribute to immune regulation beyond the intestinal niche, consistent with our observation that gut mycobiota disruption exacerbates pulmonary PCP.

While our study provides novel correlative evidence linking gut fungal alterations, serum metabolite changes, and pulmonary immune responses during PCP, several limitations should be acknowledged. First, although our FMT experiments using germ-free recipient mice provide functional evidence that gut fungal dysbiosis is sufficient to exacerbate PCP, we did not perform metabolite supplementation experiments to directly test whether specific candidate metabolites originated from gut fungi confer protection against infection. Second, in vitro assays assessing the direct immunomodulatory effects of these metabolites on immune cells were not conducted in the current study. Third, reconstitution experiments using defined fungal consortia to determine whether specific gut fungi are sufficient to restore protective immunity remain to be performed. Consequently, the proposed “gut fungi-metabolite-lung immunity axis” should be interpreted as a hypothesis-generating framework rather than a fully established causal pathway. Fourth, ITS sequencing cannot differentiate viable colonizing fungi from dead fungal spores or transient environmental fungi purely based on relative/absolute abundance data, making it difficult to definitively distinguish stably inhabited core commensal fungi from sporadic environmental fungal contaminants in the intestinal niche. Future studies incorporating metabolite supplementation, in vitro immune cell stimulation, and defined fungal reconstitution are required to definitively validate the mechanistic axis proposed here. In addition, we propose that subsequent culture-based mycobiomics, viability qPCR or metatranscriptomics are urgently needed in follow-up work to accurately validate active viable gut fungal colonizers and exclude transient contaminating/sporadic fungi, thereby remedying the inherent defects of ITS sequencing and achieving more rigorous identification of authentic functional intestinal fungi. Despite these limitations, our findings represent an important step toward understanding the role of the gut mycobiota in pulmonary host defense against *Pneumocystis*.

In conclusion, our study depicted the characteristic changes in the intestinal fungi of PCP mice and demonstrated their association with plasma metabolome disorders. In addition, we further demonstrated the role of intestinal fungi in maintaining pulmonary immune environment homeostasis following PCP, providing a potential target for PCP treatment. Our study provides novel fungal-centric evidence for the gut-lung axis, highlights potential risks associated with indiscriminate antifungal use, and provides a foundation for therapies that treat pulmonary infections by modulating the gut mycobiota.

## Electronic supplementary material

Below is the link to the electronic supplementary material.

Supplementary Material 1

## Data Availability

The data that support the findings of this study are available from the corresponding author upon reasonable request.
