# Supplementary material for: Gut mycobiota alteration contributes to the pathogenesis of Pneumocystis pneumonia
Source: J Transl Med. 2026 Jun 10;24:801. doi: 10.1186/s12967-026-08153-7 (PMC13289320; doi:10.1186/s12967-026-08153-7)
Supplement: Supplementary file 1 — Supplementary Material 1 [file 12967_2026_8153_MOESM1_ESM.docx]

| **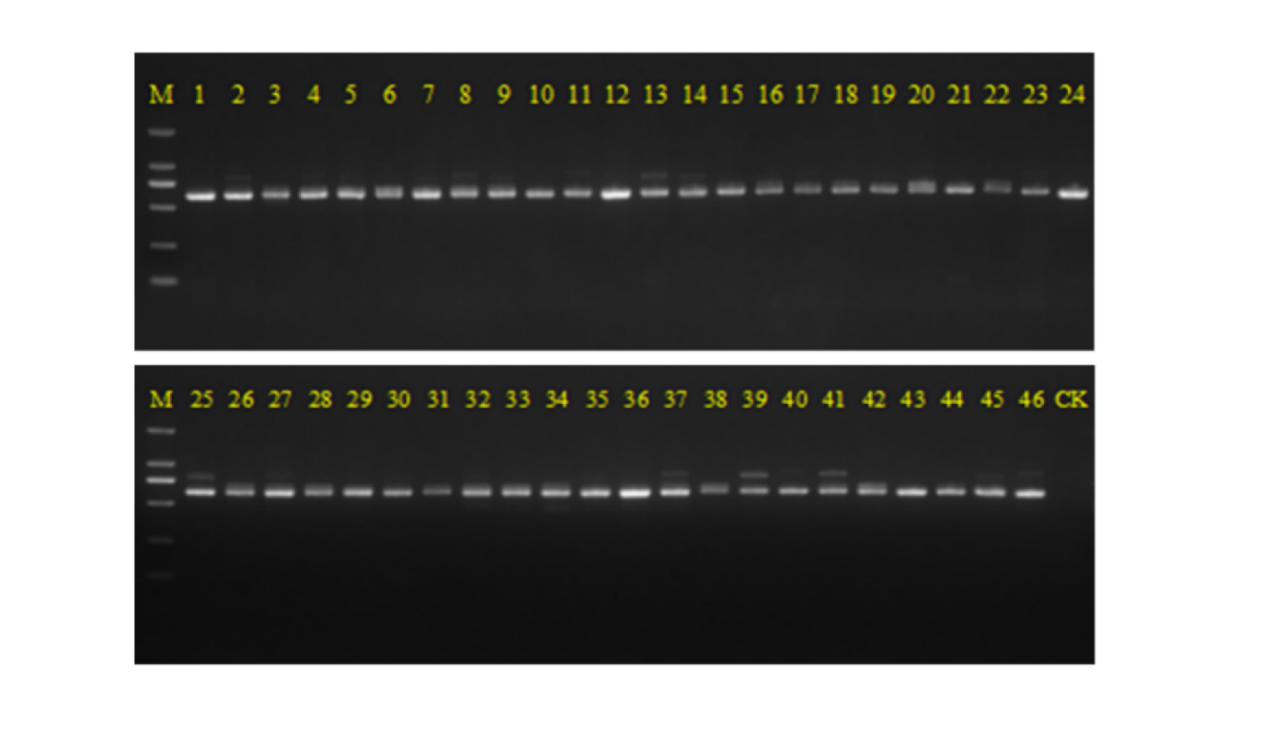** |
| --- |
| **Figure S1.** Agarose gel electrophoresis of PCR products from blank extraction control and experimental samples. No band was observed in the blank extraction control (CK), whereas all experimental samples (1-46) showed clear target bands, confirming the absence of detectable exogenous fungal DNA contamination. |
|  |
|  |
|  |
|  |
|  |
|  |
|  |
|  |
|  |
|  |
|  |
|  |
|  |
| **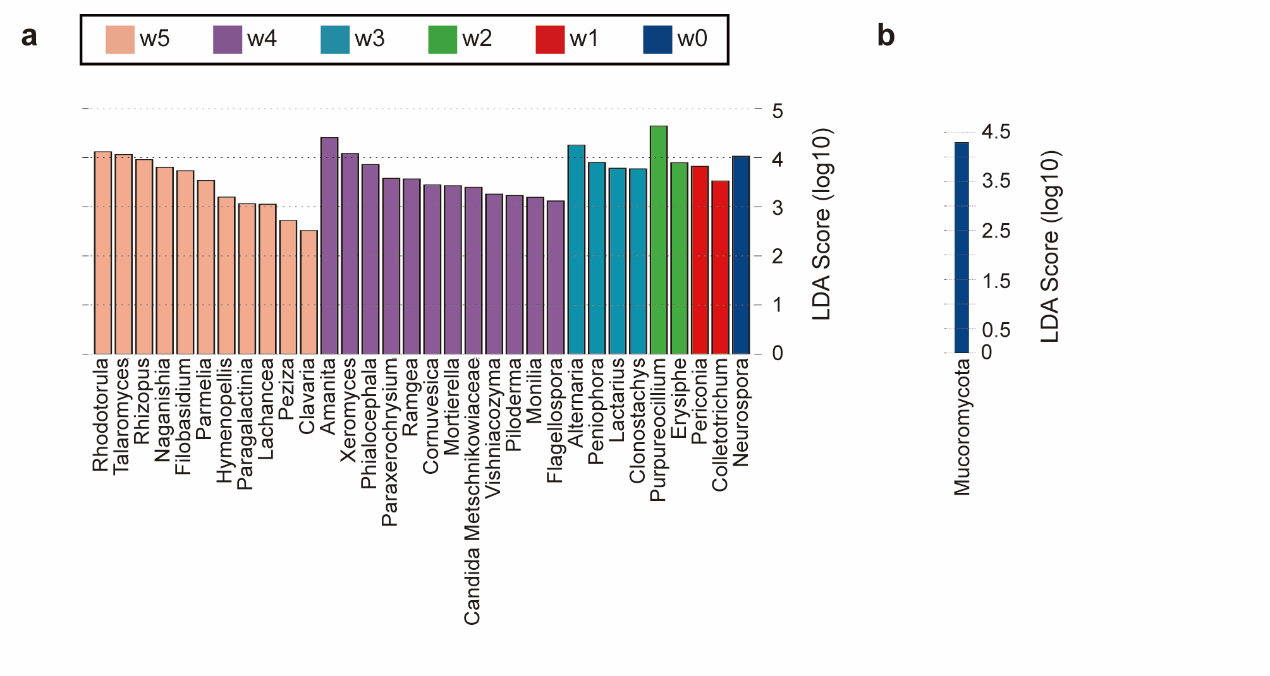** |
| **Figure S2.** Linear discriminant analysis (LDA) effect size of fungal taxa at the genus and phylum levels. Only the taxa that surpass a significant linear discriminant analysis score threshold of >2.0 and exhibit P < 0.05 in the Kruskal-Wallis rank-sum test. |
| **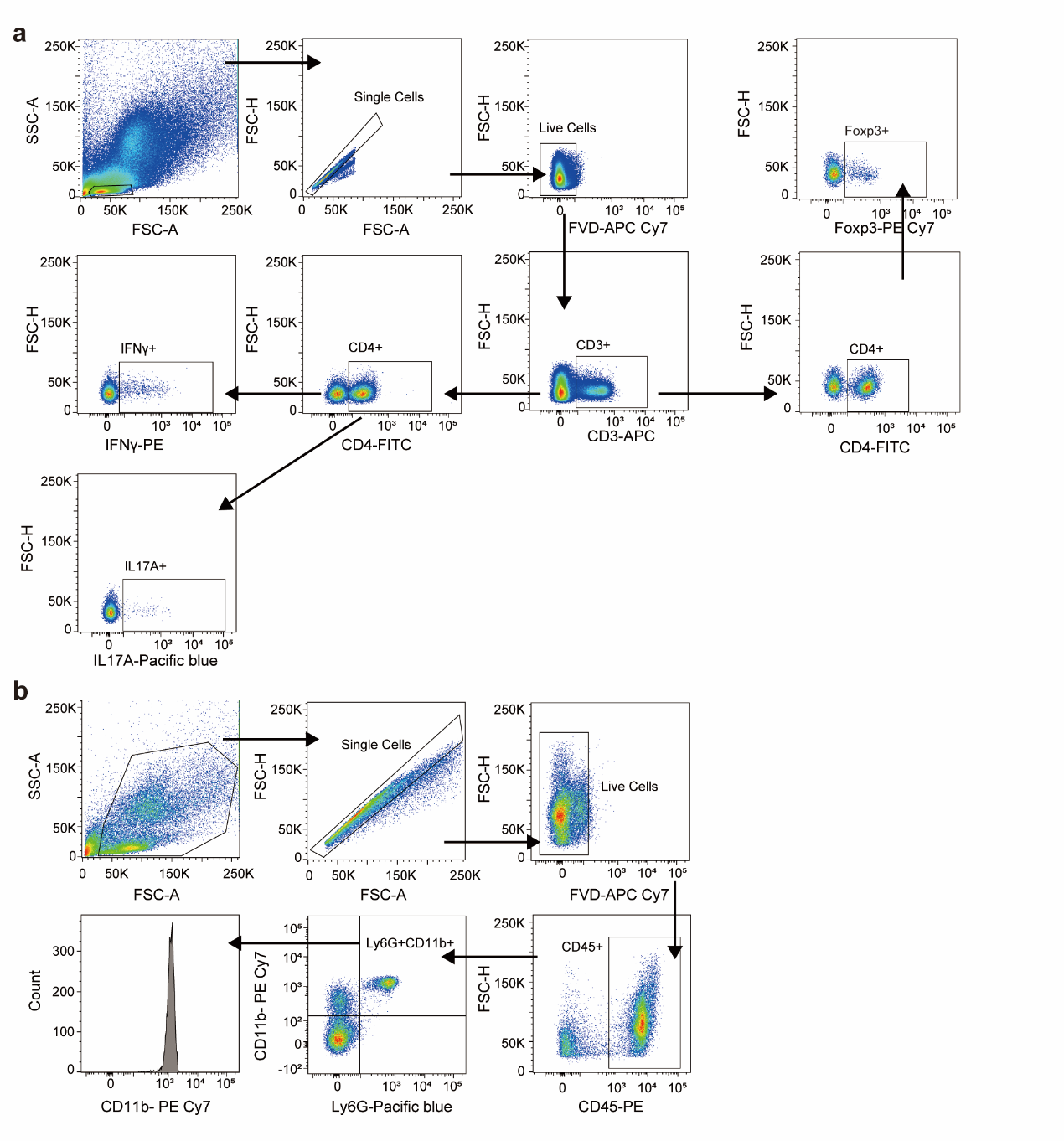** |
| **Figure S3. Flow cytometric gating strategy used to identify Th1\Th17\Treg (a), neutrophils (b).** |
|  |
| **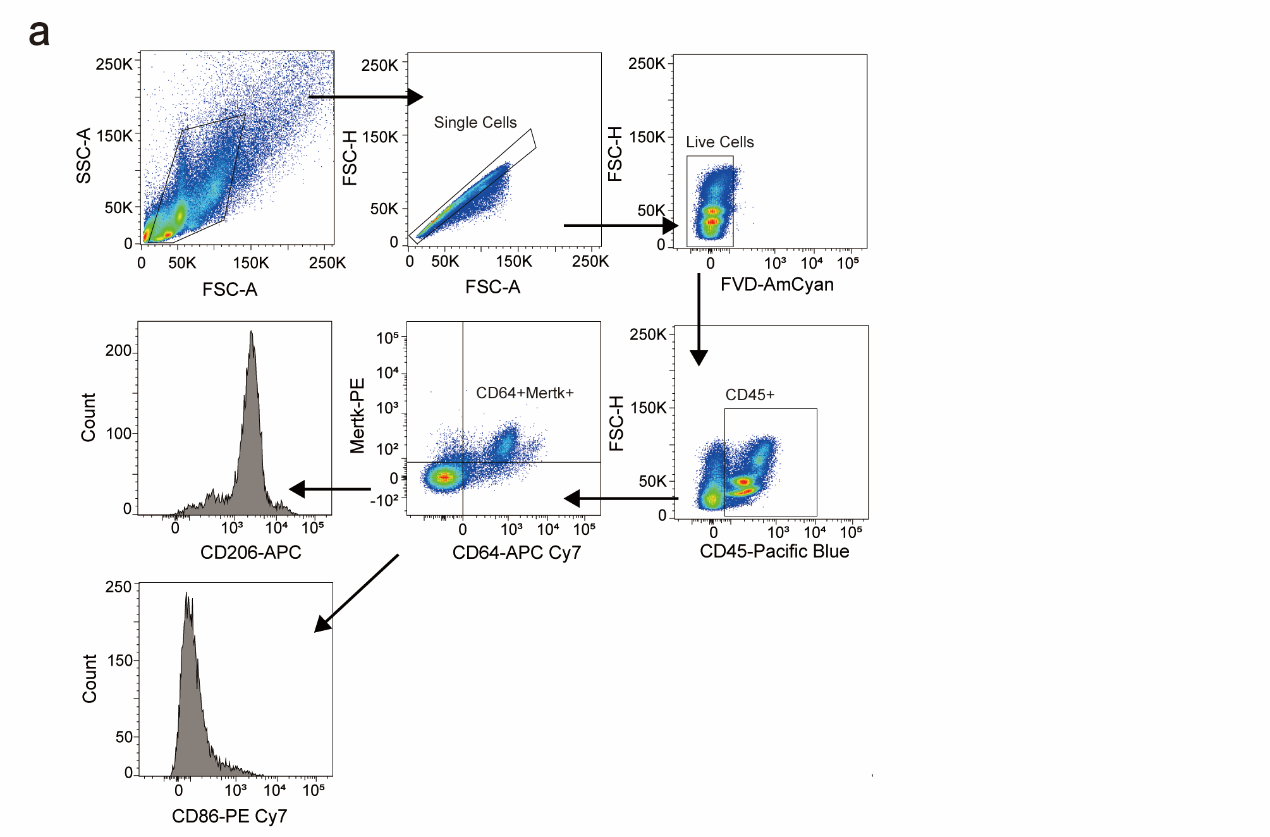** |
| **Figure S4. Flow cytometric gating strategy used to identify CD86^+^ and CD206^+^ macrophage in lung tissue.** |
|  |
| **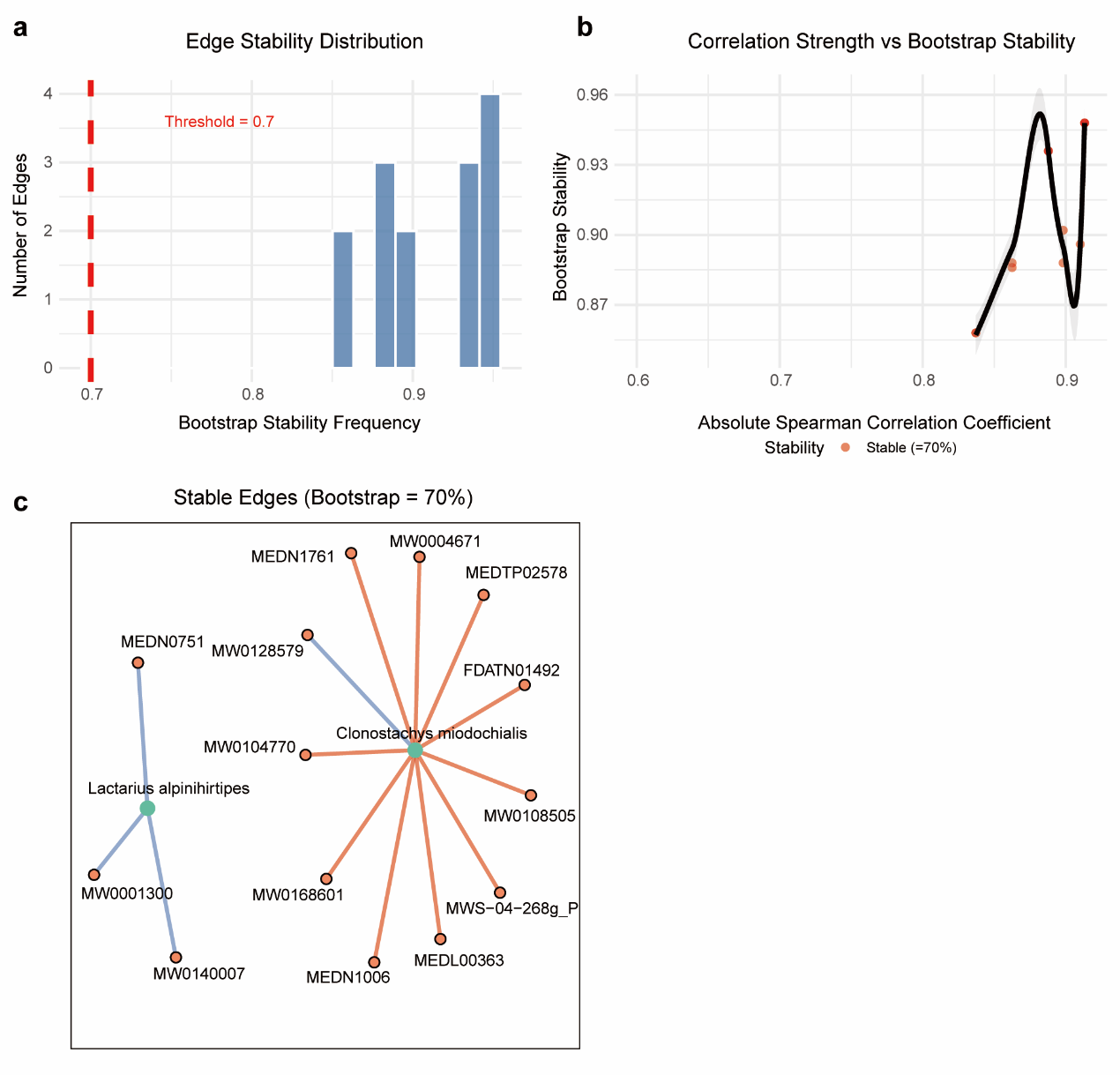Figure S5. Bootstrap validation and visualization of fungi-metabolite association networks.** (a) Histogram showing the distribution of bootstrap stability frequencies for all significant correlations. The red dashed line indicates the 70% stability threshold. (b) Scatter plot illustrating the relationship between absolute correlation coefficients and bootstrap stability for all edges. Red points denote stable edges with bootstrap stability >70%. The black LOESS curve reveals a positive trend, indicating that stronger correlations tend to exhibit higher stability. (c) Network visualization after stability filtering, showing only edges with ≥70% bootstrap stability. Green nodes represent fungal taxa, orange nodes represent metabolites, red edges indicate positive correlations, and blue edges indicate negative correlations. |
| 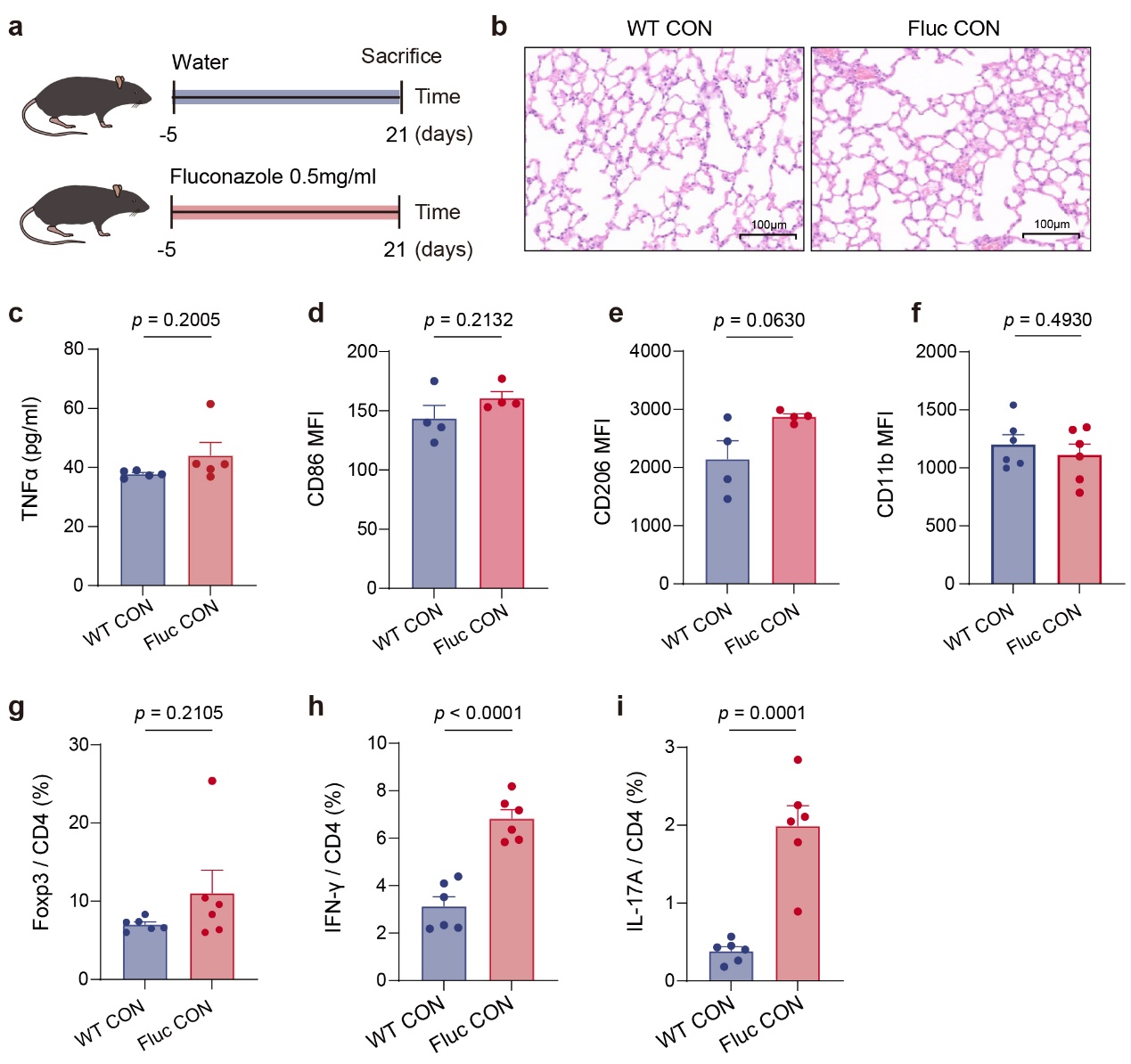 |
| **Figure S6. Direct immunomodulatory effects of fluconazole in uninfected mice.** (a) Schematic overview of the experimental design. (b) Representative H&E images. Scale bars represent 100 μm. (c) ELISA result of TNFα in mice plasma from the untreated (WT CON) and fluconazole-treated (Fluc CON) mice. (d-e) MFI of CD86 and CD206 on macrophages in the WT CON and Fluc CON mice. (f) MFI of CD11b on neutrophils in the WT CON and Fluc CON mice.  (g-i) Proportion of Tregs, Th1 and Th17 among pulmonary CD4⁺ T cells in the WT CON and Fluc CON mice. n = 4–6 per group. |
|  |
| **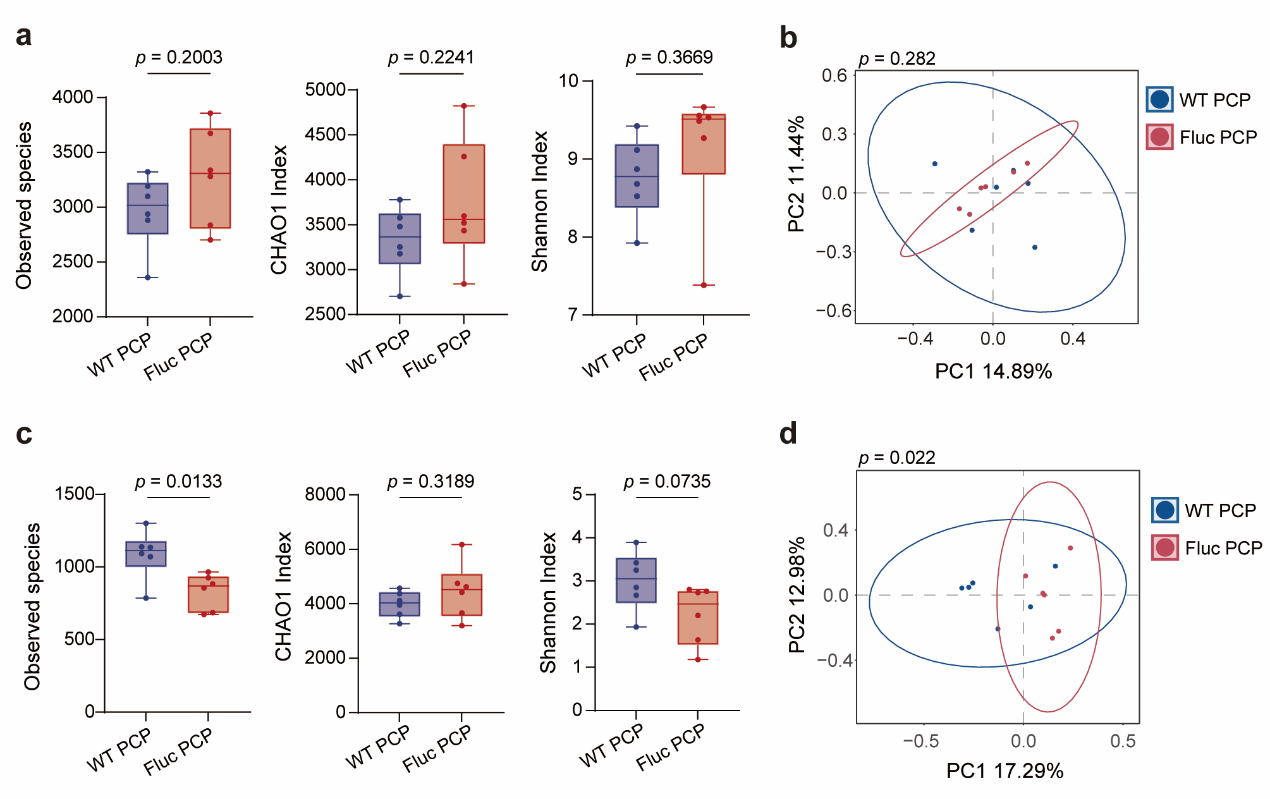** |
| **Figure S7.** α diversity (observed species, Chao1, Shannon) and β diversity (unweighted UniFrac PCoA) of bacterial (16S, **a-b**) and fungal (ITS, **c-d**) communities in WT PCP versus Fluc PCP (n=6 per group). Data are shown as median with interquartile range. |
